# Adaptive Maximal Blood Flow Velocity Estimation From Transcranial Doppler Echos

**DOI:** 10.1109/JTEHM.2020.3011562

**Published:** 2020-07-23

**Authors:** Federico Wadehn, Thomas Heldt

**Affiliations:** 1 Department of Electrical EngineeringETH Zürich8092ZürichSwitzerland; 2 Department of Electrical Engineering and Computer ScienceInstitute for Medical Engineering and Science, and Research Laboratory of Electronics, Massachusetts Institute of TechnologyCambridgeMA02139USA

**Keywords:** Transcranial Doppler ultrasound, maximal blood flow velocity, envelope tracing, signal quality assessment, intracranial pressure estimation

## Abstract

Objective: Novel applications of transcranial Doppler (TCD) ultrasonography, such as the assessment of cerebral vessel narrowing/occlusion or the non-invasive estimation of intracranial pressure (ICP), require high-quality maximal flow velocity waveforms. However, due to the low signal-to-noise ratio of TCD spectrograms, measuring the maximal flow velocity is challenging. In this work, we propose a calibration-free algorithm for estimating maximal flow velocities from TCD spectrograms and present a pertaining beat-by-beat signal quality index. Methods: Our algorithm performs multiple binary segmentations of the TCD spectrogram and then extracts the pertaining envelopes (maximal flow velocity waveforms) via an edge-following step that incorporates physiological constraints. The candidate maximal flow velocity waveform with the highest signal quality index is finally selected. Results: We evaluated the algorithm on 32 TCD recordings from the middle cerebral and internal carotid arteries in 6 healthy and 12 neurocritical care patients. Compared to manual spectrogram tracings, we obtained a relative error of −1.5%, when considering the whole waveform, and a relative error of −3.3% for the peak systolic velocity. Conclusion: The feedback loop between the signal quality assessment and the binary segmentation yields a robust algorithm for maximal flow velocity estimation. Clinical Impact: The algorithm has already been used in our ICP estimation pipeline. By making the code and the data publicly available, we hope that the algorithm will be a useful building block for the development of novel TCD applications that require high-quality flow velocity waveforms.

## I. Introduction

Due to its non-invasiveness, relatively low cost, and the possibility of repeated and continuous bedside measurements, Doppler ultrasonography is routinely used for assessing blood flows in different organs [1]. In neuro-monitoring, transcranial Doppler (TCD) ultrasonography has been suggested for the diagnosis of stenosis [2], vasospasms [3], and large cerebral vessel occlusions, which are characterized by alterations in blood flow velocities and waveform morphologies [4], [5]. Recent work also suggests that TCD ultrasonography, combined with minimally-invasive blood pressure measurements, can be used for monitoring cerebral autoregulation [6], [7] and for obtaining non-invasive estimates of intracranial pressure (ICP) [8]–[11]. In most neuromonitoring applications, the quantity of interest is the maximal blood flow velocity waveform, which together with measurements or assumptions on the vessel geometry, can be used to estimate volumetric blood flow.

### A. Pulsed Doppler Ultrasonography

**FIGURE 1.**
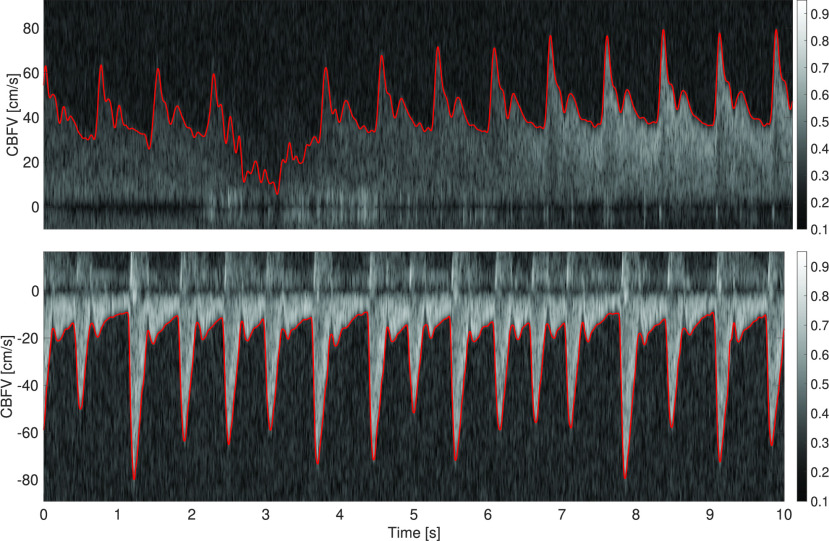
Top: Doppler spectrogram obtained by insonating the M1 segment of the middle cerebral artery. The frequency shift is converted to a velocity, such that the cerebral blood flow velocity (CBFV) is expressed in cm/s. The spectrogram shows varying levels of intensity and a signal loss between 2 s to 4 s. The algorithmically determined envelope is marked in red. Bottom: Doppler spectrogram and envelope obtained from the internal carotid artery.

Pulsed Doppler ultrasonography is commonly employed to obtain blood flow velocity measurements [12]. In pulsed Doppler ultrasound, short acoustic pulses with a fixed carrier frequency $f_{c}$ are emitted toward a target segment of a blood vessel. In the insonated target volume, the acoustic pulses are reflected by an ensemble of scatterers (red blood cells), moving at different velocities. Reflection of the acoustic pulses from moving red blood cells leads to characteristic frequency shifts in the ultrasound echo signal (see Section II-A). The received echo signal is sampled, digitized, demodulated, and split into its in-phase and quadrature components that are denoted by the (complex-valued) time-domain signal IQ[$\cdot $]. The time-varying frequency content of the demodulated echo signal and therefore the velocity distribution of the scatterer ensemble is then obtained by spectral analysis of the IQ[$\cdot $] signal. We denote the time-frequency (or time-velocity) spectrogram as SP$[\cdot, \cdot]$, where the first argument is time and the second frequency or equivalently velocity, as in Fig. 1.

The envelope of the Doppler spectrogram (see Fig. 1) corresponds to the maximal flow velocity, which is the TCD quantity of greatest clinical relevance [12]. For example, Doppler ultrasound indices – such as the pulsatility or resistivity indices [13] – are defined in terms of the maximal flow velocity. A further advantage is that for measuring the maximal flow velocity, it is sufficient that the sample volume contains the center segment, where the velocity of the scatterers is the highest in laminar flow. By contrast, to obtain the mean flow velocity it would be necessary that the cross-sectional area of the sample volume matches the blood vessel.

### B. Literature Review

The TCD spectrogram tracing algorithms employed in commercial ultrasound systems are commonly proprietary and integrated into the software of ultrasound machines. Due to the desire for immediate visualization of maximal flow velocity waveforms in the clinics, these algorithms are mainly tailored to (near) real-time operation [14], [15]. However, for an increasing number of applications as for example model-based estimation of ICP [11], [16], high-quality flow velocity waveforms are required, whereas real-time operation is not a hard constraint. The algorithms in [8] and [11], for example, return an estimate of mean ICP every minute. The possibility of offline processing consequently paves the way for more elaborate algorithms for obtaining high-quality maximal flow velocity waveforms.

In the academic literature, one can mainly identify three types of approaches for maximal flow velocity estimation. First, classical methods based on noise threshold estimation [17] and variants thereof [18]. The modified threshold crossing method (MTCM) evaluated in [19], for example, compares a group of spectral samples (selected from a column of the spectrogram SP$[\cdot, \cdot]$), starting from the noise-end of the spectrogram, with a preset threshold. Once a sufficient number of samples in the group exceed the threshold, the maximal flow velocity is set as the highest frequency bin in the group. This approach is popular due to its simplicity, but its performance depends on the choice of the preset threshold and the intensity distribution of the spectrogram samples at each time point. The second type of approaches employ image processing techniques, such as edge-detection and edge-following [20], [21]. The third type of approaches use parametric waveform models that are fit to single frequency bins of the spectrogram [22].

An example of a supervised edge-detection and edge-following algorithm was presented in [21], where the authors propose a two-step approach: In the first step, the intensity histogram of each spectrogram sub-window is compared, via the Kullback–Leibler divergence, to the intensity distribution of a segment that is known to contain noise only. On the thresholded image, a standard edge enhancement algorithm (difference of Gaussians) is run. In the second step, the obtained envelope is modeled as a white noise acceleration model and is filtered using a Kalman filter. Similarly, in [20] an algorithm is proposed that uses a nonlinear Laplace edge detector, whose result is then refined by eliminating spurious edges via the flood-fill algorithm. Since at each time point (that is, for each column of the spectrogram) there is only one maximal flow velocity and since the flow velocity must fulfill certain physiological constraints, physiological sanity-checks can be put in place to avoid spurious edges and obtain better envelopes, as discussed in Section II.

By contrast, in [22] the spectrogram is treated as multiple time series (one for each frequency bin) and a parametric model is fit to each time series. In this model-based approach, a Gamma function is used as a parametric model for the blood flow velocity waveform. Such parametric approaches, based for instance on Gamma functions or Gaussian mixtures, will often produce smooth-looking envelopes [22]. However, a drawback of such analytical functions is that they lack a physiological basis and for distal vessels the “rounded” beat wavelet lacks details such as the dicrotic notch and other fiducial points. Furthermore, different arteries have quite a different waveform morphology, as shown in Fig. 1, such that each vessel would require a tailored parametric model.

In this paper, we propose an offline algorithm for estimating maximal blood flow velocities from Doppler spectrograms. The proposed algorithm is calibration-free and fully unsupervised. Its main novelty is a feedback loop between a signal quality index and an image segmentation algorithm. In addition to parameter selection, the signal quality algorithm provides a beat-by-beat measure of confidence for the reported blood flow velocity waveform. This can be useful when passing the blood flow velocity signal to downstream processing algorithms and when interpreting derived quantities such as the pulsatility index or noninvasive ICP estimates. The code and the data is available on the IEEE DataPort [23].

## II. Estimation of the Maximal Flow Velocity

**FIGURE 2.**
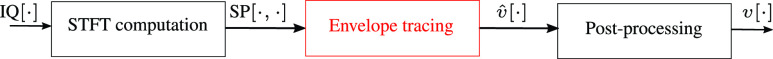
Maximal flow velocity estimation pipeline. The demodulated echo signal IQ$[\cdot]$ is converted to the spectrogram SP$[\cdot, \cdot]$ via the short-time Fourier transform (STFT). Then, the maximal flow velocity waveform (envelope of the spectrogram) $\hat {v}[\cdot]$ is computed, and finally the flow velocity is filtered to remove noise and outliers.

The starting point of our algorithm is the demodulated time-domain echo signal IQ[$\cdot $]. To obtain the maximal flow velocity waveform from the demodulated echo signal we first compute its spectrogram SP[$\cdot, \cdot $]. Then, we run an envelope tracing algorithm, and finally perform post-processing steps on the detected envelope to enhance the quality of the maximal flow velocity waveform (Fig. 2).

### A. Spectrogram Computation

Low-frequency reverberations due to vessel wall movements are a major source of disturbance when measuring blood flows [24]. These disturbances can be removed from the IQ signal with a high-pass filter (so-called wall filter), for which we used a second-order Butterworth filter with a cutoff frequency of 100Hz. We obtain the spectrogram by computing the power spectral density on small windows (e.g., 10 ms) of data with 75% overlap. Subsequently, we apply a log-compression to reduce the dynamic range of the intensity values. Then, we divide by the maximal intensity of the spectrogram and in addition set all negative log-intensity values to zero. The intensity values of the transformed spectrogram thereby fall into the range [0, 1] (see Fig. 1 for a color scale.) Finally, the frequency shift $\Delta f$ is converted to the velocity $v$ via

\begin{equation*} v = \frac {\Delta f \cdot c}{2 f_{c} \cos (\alpha)}, \tag{1}\end{equation*}

where $c = 1540$ m/s is the assumed (average) speed of sound in tissue, $\alpha $ the angle between the direction of the ultrasound beam and the flow velocity vector, and $f_{c} = 1.75$ MHz the carrier frequency of the ultrasound pulse. The sampling frequency of the demodulated echo signal (given the settings used in our acquisition) was $f_{s}^{{\mathrm {Echo}}} =6.944$ kHz. Therefore, the range of detectable frequency shifts is $[-f_{s}^{{\mathrm {Echo}}}/2, + f_{s}^{{\mathrm {Echo}}}/2]$. Taking the maximally detectable positive frequency shift of $+f_{s}^{{\mathrm {Echo}}}/2=3.472$ kHz, Eq. (1) results in a maximally detectable flow velocity of about 152 cm/s, given our chosen acquisition parameters. Since we had no information on the insonation angle, we set $\alpha = 0^\circ $ for all recordings, which is commonly a reasonable assumption for the middle cerebral artery (MCA) and internal carotid artery (ICA). In addition, many applications such as the non-invasive estimation of ICP [8], [11] require only a scaled version of the maximal cerebral blood flow velocity. In the following, we describe the algorithm used to obtain maximal blood flow velocity estimates from the Doppler spectrogram.

### B. Overview of the Flow Velocity Estimation Algorithm

High intensity pixels in the TCD spectrogram (see Fig. 4) indicate the presence of a collection of scatterers flowing through the sample volume with a particular velocity relative to the probe, whereas noise regions are characterized by being (significantly) darker. Therefore, one approach to find the maximal flow velocity is to perform a binary black-and-white (B&W) segmentation of the spectrogram to separate signal (white) from noise (black). The goal then is to find for each time step the maximal flow velocity, that is, the pixel with the highest flow velocity that still belongs to the signal-carrying part of the spectrogram.

**FIGURE 3.**
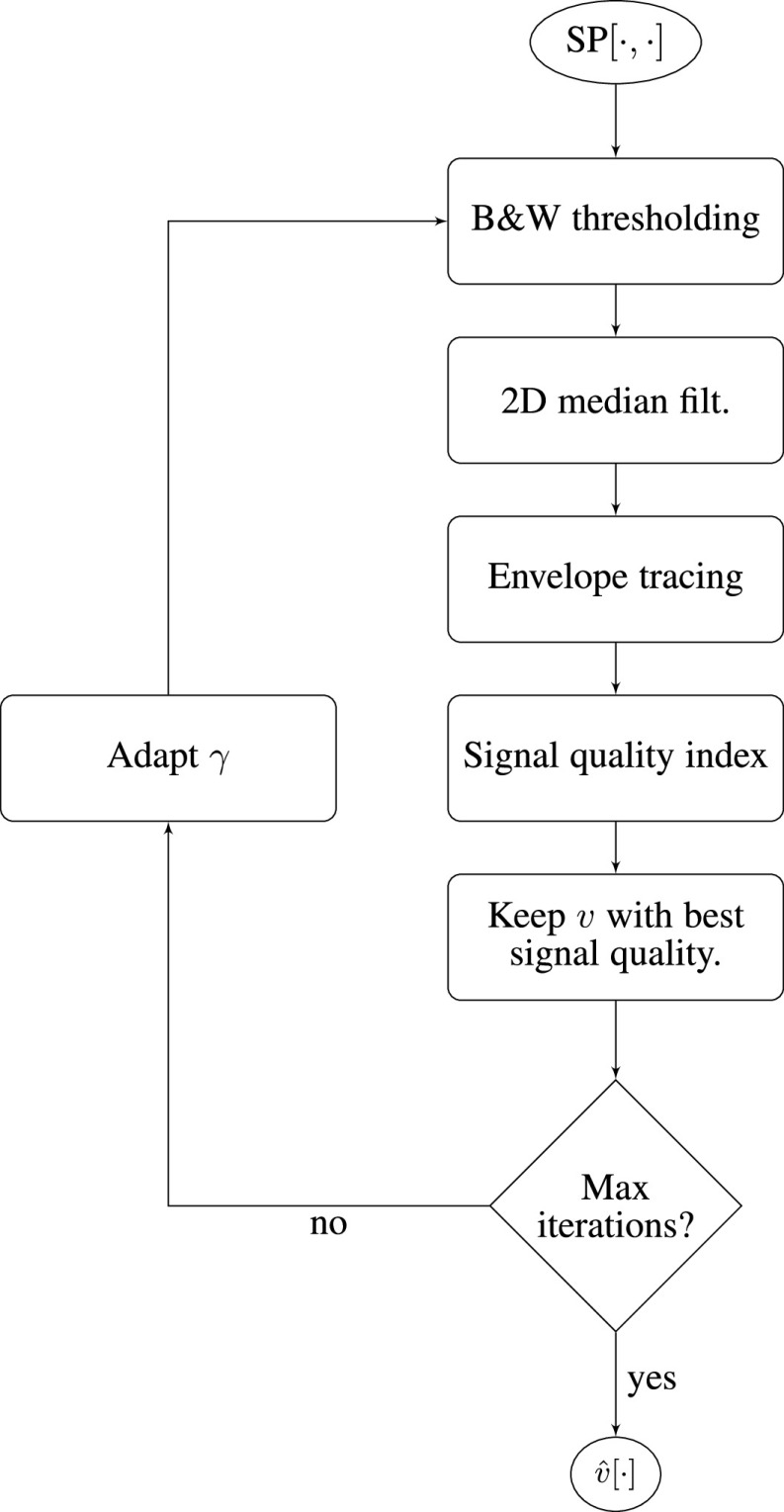
Flow chart of the envelope tracing (maximum flow velocity estimation) algorithm, representing the red box in Fig. 2.

**FIGURE 4.**
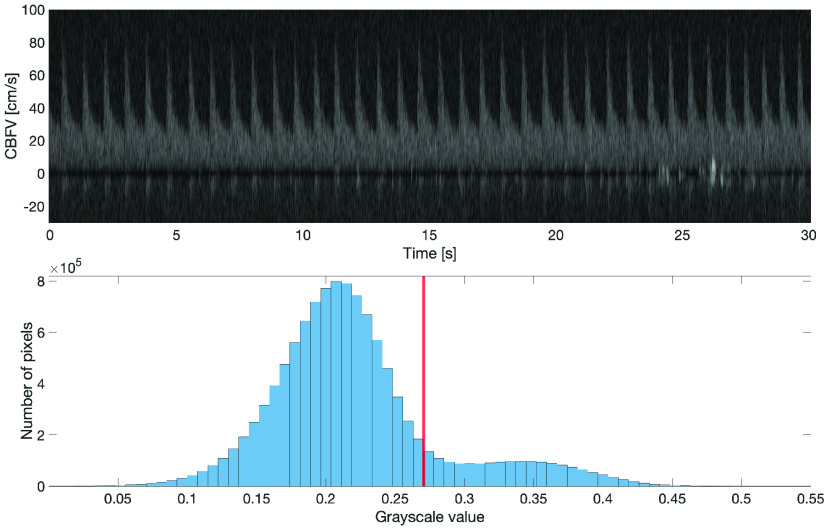
Histogram of grayscale values of Doppler spectrogram. The red bar indicates the Otsu threshold that separates the signal (above threshold) from noise.

The flow chart in Fig. 3 provides a high-level overview of the maximal flow velocity estimation algorithm. To obtain the maximal flow velocity waveform, we first pick a candidate intensity threshold $\gamma $ to perform a binary segmentation of the spectrogram ($\text{B}\&\text{W}$ thresholding) and then run a 2D median filter to remove speckle noise. Subsequently, we perform an envelope tracing step and finally assess its signal quality through a series of physiological sanity checks and template matching. If the signal quality of the candidate flow velocity estimate $v$, obtained using the candidate threshold, exceeds the quality of the best flow velocity waveform obtained so far, we set the current flow velocity waveform as the current best waveform $\hat {v}$. Once the maximal number of preset iterations is reached, that is, all grayscale thresholds have been tested, we terminate the algorithm and return the maximal flow velocity waveform $\hat {v}$ that has the highest signal quality. In the following, we describe the image segmentation and envelope tracing approaches in more detail.

### C. Binary Segmentation of the Spectrogram

Figure 4 shows a histogram of grayscale values in the range [0, 1] from a 30 s spectrogram segment. Since there is considerable overlap between the grayscale values of the signal and the noise, using a fixed, predetermined segmentation threshold to binarize the spectrogram and then taking the maximal flow velocity at each point in time would not result in an acceptable maximal flow velocity estimate. Given the bi-modal shape of the histogram, however, we can expect a reasonable separation between signal and noise by binary image segmentation, though the optimal threshold remains to be determined on a window-by-window basis. To obtain an initial estimate of the segmentation threshold, we employ Otsu’s method [25], which performs binary clustering of the image’s binned grayscale values. Otsu’s method uses the same cost function as the k-means algorithm [26], namely the minimization of the within-class variance. However, unlike k-means clustering, it uses the fact that each pixel is a scalar (grayscale) value, which allows for the method to perform an exhaustive search for an optimal segmentation threshold $\gamma ^{*}$.

Our final objective, however, is not the minimization of the within-class variability, but to perform a binary image segmentation such that the recovered envelope of the spectrogram represents as closely as possible the true maximal flow velocity. In fact, the spectrogram envelope obtained solely from binary segmentation based on Otsu’s method tends to be overly flat, that is, the peak velocity tends to be underestimated. Nonetheless, the optimal grayscale threshold computed with Otsu’s method provides a good starting point, and – in our experience – small adaptations of this threshold oftentimes lead to accurate spectrogram envelope estimates.

In our proposed algorithm, we therefore perform a grid search around the threshold $\gamma _{Otsu} = \gamma ^{*}$ computed via Otsu’s method. We can, for instance, use the following segmentation thresholds

\begin{align*} \gamma \in \{0.9 \gamma _{{\mathrm {Otsu}}}, 0.95 \gamma _{{\mathrm {Otsu}}}, \gamma _{{\mathrm {Otsu}}}, 1.05 \gamma _{{\mathrm {Otsu}}}, 1.1 \gamma _{{\mathrm {Otsu}}}\} = \Gamma. \\ \tag{2}\end{align*}

**FIGURE 5.**
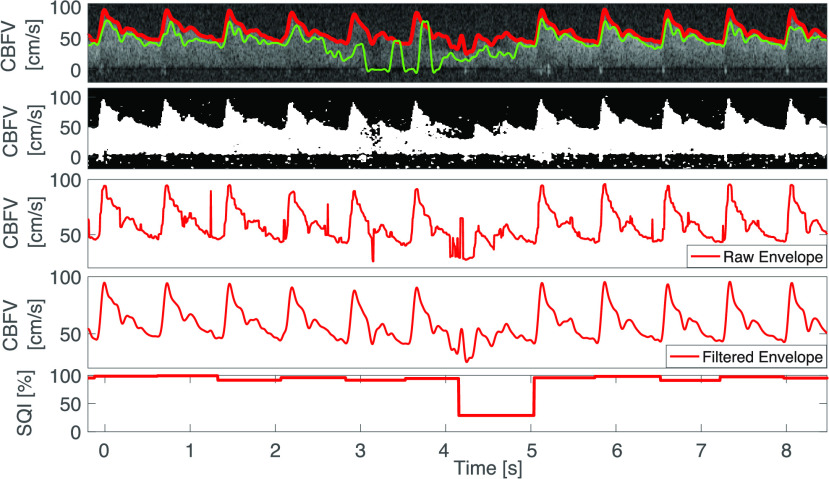
First row: Automatic envelope tracing by proposed algorithm (red) and MTCM (green). Second row: Binarized spectrogram. Third row: Raw envelope signal with some spikes. Fourth row: Filtered final output. Fifth row: Signal quality index.

Then, the spectrogram is binarized with each threshold (see Fig. 3). Since the binarized images usually still contain some speckles (see second row of Fig. 5), these residual speckles should be removed before performing the envelope detection. Given the shape of the speckles, this is done with a 2D median filter with a kernel whose horizontal length is 0.03 s and whose vertical length is 5 cm/s. For obtaining a good segmentation threshold via Otsu’s method, it is beneficial to choose an appropriate region of the spectrogram on which the grayscale threshold is computed. Ideally, the two classes – the signal and the noise – should be balanced [26]. Since both negative flow (e.g., in the ICA) and positive flow (e.g., in the MCA) can occur (see Fig. 10), we compare the energy in the positive spectrogram region (5 cm/s up to 125 cm/s) to the energy in the negative region (-125 cm/s up to −5 cm/s) and take the region with the highest energy (sum of pixel intensities) as the signal-containing region. The spectrogram window starts at ±5 cm/s in order to exclude the stop-band region of the wall filter; see dark region around 0 cm/s in Fig. 1. Note that with this approach, we cannot detect reverse flow, that is, flow velocities that range from negative to positive values.

**FIGURE 6.**
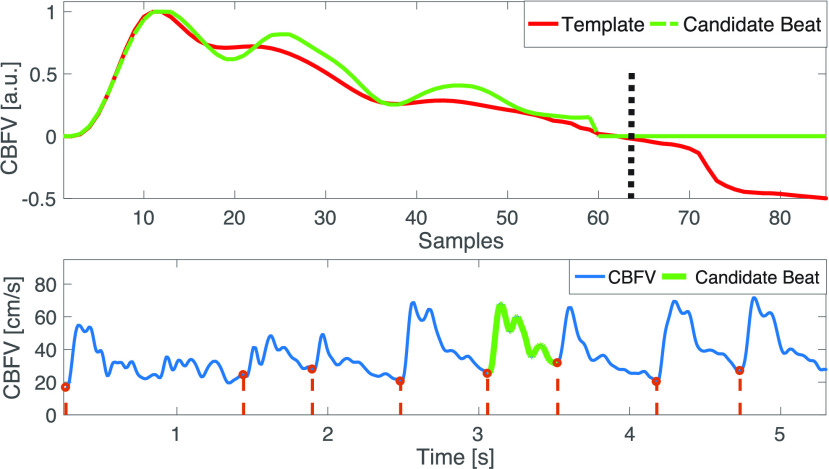
Beat-by-beat signal quality assessment of the maximal blood flow velocity waveform by template matching. The MSE is computed on the first 75 % of the template’s length (zero to black dashed bar). The candidate beat (shown in green) has a high quality as it matches well to the template.

**FIGURE 7.**
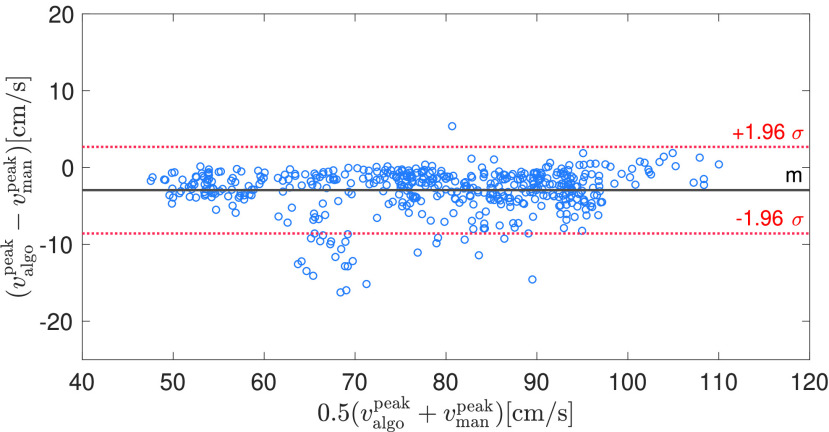
Bland-Altman plot for estimated (with our algorithm) vs manually-traced peak systolic velocity in 16 MCA recordings from healthy subjects.

**FIGURE 8.**
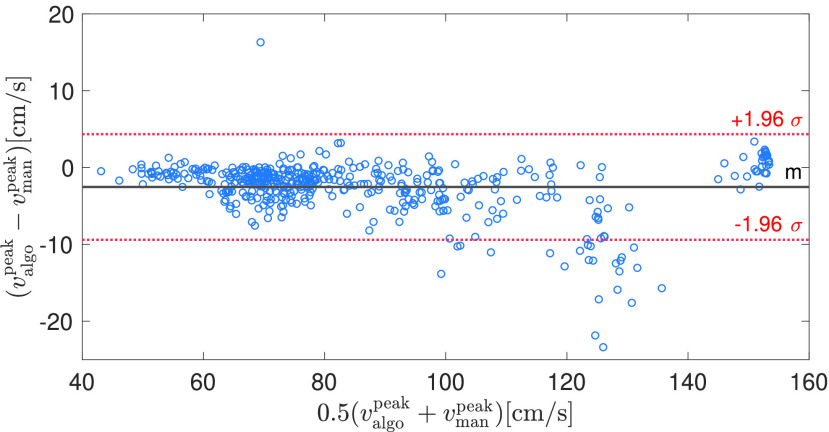
Bland-Altman plot for estimated (with our algorithm) vs manually-traced peak systolic velocity in 16 MCA and ICA recordings from neurocritical care patients. Note that the neurocritical care patients have a wider range of peak systolic velocities compared to the healthy subjects.

**FIGURE 9.**
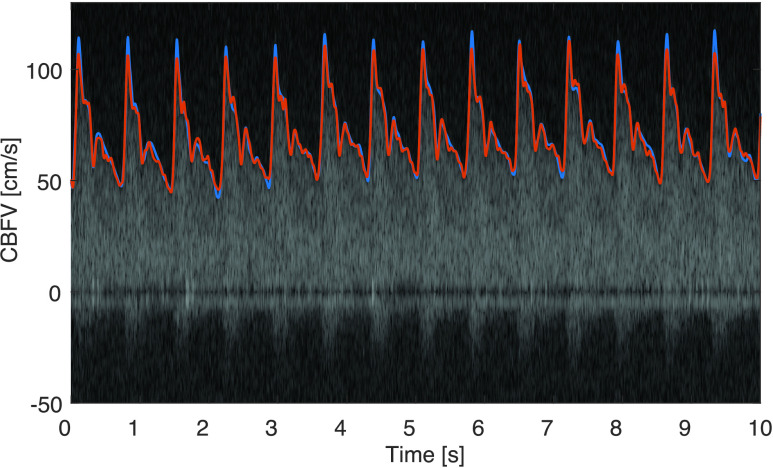
Comparison of manually traced (blue) and algorithmically determined (red) maximal cerebral blood flow velocity. Note that the peak-systolic velocity is slightly underestimated.

**FIGURE 10.**
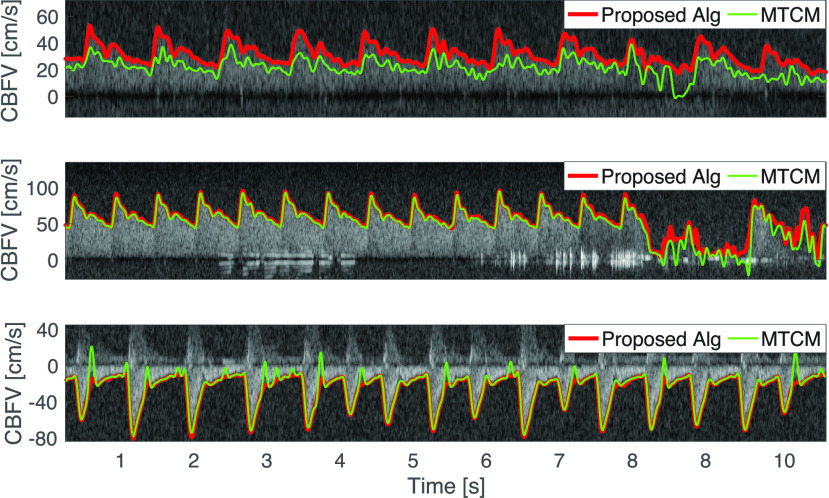
Maximal blood flow velocity determined with proposed (red) and MTCM algorithm (green). Data obtained from the MCA of a healthy subject (top), the MCA of a neurosurgical patient with elevated flow velocity (center), and the ICA of a neurosurgical patient (bottom). For all three data segments the visual assessment for the proposed algorithm was “good”, whereas for the MTCM algorithm only the center and bottom plot are considered to have a good tracing quality, whereas the top plot has a bad quality.

### D. Spectrogram Envelope Tracing

Given the set of binarized spectrograms (see second row Fig. 5 for an example of a binarized spectrogram), the goal is to extract the maximal flow velocity in a column-by-column (time steps) fashion. In the following, we assume that the sampling rate is 217 Hz and that the velocity resolution is $\Delta v = 0.29 $ cm/s. Our algorithm extracts the first 5 candidate velocities (white pixels) $v[k]^{(1) },\ldots, v[k]^{(5) }$, starting from the maximal velocity of 152 cm/s in each column. These candidate velocities, starting from the highest, are subject to two physiological sanity checks

1) The majority of the 40 pixels ($\approx 12$ cm/s) below the candidate pixel (velocity) have to be white (above threshold). This avoids mistaking isolated speckles as signal.
2) The velocity estimate $\hat {v}[k]$ cannot deviate by more than 30 cm/s from the mean value of the velocity estimates of the previous three time steps ($\approx 14$ ms) $\hat {v}[k-1], \hat {v}[k-2]$, and $\hat {v}[k-3]$. This avoids isolated outliers.

If none of the 5 candidate velocities passes the sanity checks, the velocity $\hat {v}[k]$ is set to the previous value $\hat {v}[k-1]$. However, if no candidate velocity fulfils the sanity checks for more than 20 consecutive time steps, the maximal candidate velocity is taken regardless. These sanity checks are not very restrictive and are aimed at flagging only obvious outliers. A more stringent signal quality assessment follows in the next step.

### E. Signal Quality Assessment

Each candidate maximal flow velocity waveform, obtained using a specific grayscale threshold $\gamma \in \Gamma $, is evaluated on a beat-by-beat basis via a signal quality assessment algorithm. The signal quality assessment algorithm is based on thresholding amplitude and duration features as well as template matching to assess similarity between candidate beats and a template obtained from the data. This approach is similar to the method we first presented in [27] and to the recent signal quality paper by the Neural Analytics Inc (Los Angeles, CA, USA) group [28].

The first step of our signal quality assessment algorithm is to detect beat-onsets using the algorithm from [29]. This algorithm was originally developed for arterial blood pressure waveforms, but can nonetheless be used to detect beat onsets in flow velocity waveforms by scaling the input waveform such that its range matches the range of values expected for arterial blood pressure (e.g., average peak value around 140 mmHg). Each beat is then subjected to the following physiological sanity checks

1) Systolic maximum >30 cm/s.
2) Difference between maximum and minimum flow velocity in each beat $> 20$ cm/s.
3) Beat duration $>0.25$ s and < 2 s.
4) 0.5 (median beat duration) < beat duration < 2 (median beat duration).

The rationale behind conditions 1 and 2 is that for the cerebral vessels considered in this work the presumed insonation angle is $ < 20^{\circ }$. In this case, if we exclude large vessel occlusions, the amplitude and pulsatility of the flow velocity needs to be sufficiently large to be valid. Very low amplitudes and lack of pulsatility, generally indicate signal loss (see Fig. 6). Condition 3 is based on the assumption that the minimal heart rate is above 30 and below 240 beats per minute, a condition that is not overly restrictive. Condition 4 excludes excessively short or long beats, that may indicate the presence of false positives (multiple onsets detected in the same beat) or false negatives (missed beats). A similar duration-based outlier detector was used in [30] for improving their cerebral blood flow velocity pulse-onset detector.

In the next step we compute for each candidate maximal flow velocity waveform a beat template by taking the median (with zero-padding of short beats) of all beats of the 1 min segment that have not been rejected by the physiological sanity checks described above. Then, each beat that has not been flagged by the physiological sanity checks is assessed in terms of the mean squared error (MSE) between the template (computed using the candidate waveform under examination) and the candidate beat. To account for heart rate variability, which mainly affects the diastolic phase of a flow velocity wavelet, the mean squared error (MSE) is only computed on the first 75% of the template beat duration (Fig. 6). If the normalized MSE is above 30 %, the beat is flagged as an artifact. Since in our recording setup we did not expect large heart rate variability on 1 min segments, we omitted any linear or non-linear (such as dynamic time warping) beat alignment.

We define the artifact-index as the percentage of beats that were labeled as artifacts. For each 1 min segment we then choose the candidate flow velocity waveform with the smallest artifact-index as our final estimate. Note that, in addition to allowing us to select a flow velocity waveform (corresponding to a specific grayscale threshold), the signal quality assessment algorithm provides us with a measure of confidence, a signal quality index (0 % - 100 %) for each beat (Fig. 5 bottom panel). The signal quality index is computed as one minus the normalized relative MSE between each beat and the template. Finally, the remaining step to obtain high-quality flow velocity estimates is post-processing.

### F. Post-Processing of Blood Flow Velocity

Since rapid oscillations in the maximal flow velocity waveform can be attributed to noise and artifacts, the resulting signal is filtered with a 4th-order Butterworth filter with a cutoff frequency $f_{c} = 16$ Hz and additionally, with a 1D-median filter with a length of 3 samples (≈ 14 ms) to remove potential outliers (Fig. 5, third and fourth panel).

### G. Summary

To obtain a maximal flow velocity waveform from a received pulsed echo signal, we perform the following steps

1) Compute spectrogram from demodulated echo signal.
2) Compute a candidate threshold for binary segmentation via Otsu’s method on an appropriately chosen segment of the spectrogram.
3) Trace the maximum flow velocity waveform on multiple binarized spectrograms that are obtained using different segmentation thresholds.
4) Compute the signal quality for each waveform.
5) Choose the flow velocity waveform with the highest signal quality.
6) Perform low-pass and median filtering as a post-processing step.

### H. Real-Time Implementation of the Algorithm

The current implementation of the envelope tracing algorithm works by splitting the recording into one-minute segments to account for potential changes in the echo intensity over time. However, the segment length can be reduced to, e.g., 10 s to allow a near-real-time implementation of the envelope tracing algorithm. In addition, to reduce computational costs, one could start with a given segmentation threshold that is only adapted (on a segment-by-segment basis) if the signal quality of the retrieved maximal flow velocity waveform is below a certain threshold.

## III. Materials and Evaluation Metrics

### A. Human Subject Data and Study Protocols

The TCD data were collected from healthy volunteers at Massachusetts Institute of Technology (MIT) and from patients in neurocritical care at Boston Medical Center (BMC). Data collection occurred between 2016 and 2020, was approved by the MIT and BMC Institutional Review Boards, and informed consent was obtained from the subjects directly at MIT or from the patients or their legally authorized representatives at BMC. The healthy subject population consisted of six subjects in the age range of 25–45 years with no known neurological disorders. From these, we obtained in total 16 TCD recordings from the middle cerebral artery (MCA) with a total duration of about two hours. The neurocritical care patient population consisted of 12 patients, aged 23–74 years, presenting with conditions such as traumatic brain injury (TBI), hydrocephalus, and intraparenchymal or subarachnoid hemorrhage. Details of the clinical data collection are provided in Jaishankar *et al.* [31]. From the neurocritical care patient population, we included in total 16 TCD recordings from either the MCA or the internal carotid artery (ICA) with an aggregate duration of 2 h and 40 min.

We used the portable Philips CX-50™ ultrasound system (Philips, Andover, MA) with a S5-1 ultrasonic transducer with a frequency of 1.75 MHz. The spectrogram was computed by a short-time Fourier transform of the demodulated echo signal on overlapping windows with a 75% overlap. The shift between overlapping windows was 1/217 s, resulting in a sampling frequency of 217 Hz of the blood flow velocity waveform. The sampled echo data was retrieved from the internal memory of the CX-50 ultrasound system and processed using the Image Processing Toolbox™ in MATLAB 2018a (The Mathworks, Natick, MA).

To obtain MCA cerebral blood flow velocity recordings, the sonographer positioned the ultrasound probe over the temporal ultrasound window and mostly targeted the M1 segment of the MCA. This approach results in a dominant blood flow towards the probe, which is registered as a positive flow velocity. To obtain ICA blood flow velocity recordings, the sonographer positioned the ultrasound probe on the throat, pointing towards the base of the skull with an acute angle with respect to the vessel direction. This results in a blood flow away from the probe, and hence negative blood flow velocity. In both cases, the ultrasound probe was manually stabilized, and therefore small hand or head movements often led to signal deterioration or even signal loss (Fig. 1, top).

### B. Manual Spectrogram Tracing and Rating

To obtain a quantitative assessment of the performance of our envelope tracing algorithm, we developed a custom-made MATLAB program for manually tracing TCD spectrograms. In each recording, we randomly selected a spectrogram segment. If the quality of the spectrogram in that segment was sufficient to visually detect an envelope, we manually traced 30 heart beats. Otherwise we randomly selected another segment and again assessed if it was possible to visually detect an envelope. The metrics used for the quantitative evaluation of our algorithm, compared to the manual traces, are described in Section III-C.

For a qualitative assessment that does not rely on manually-traced spectrograms we rated each recording using the three categories (good, acceptable, and bad). We used the following rules for categorizing each recording: An envelope trace was seen as ‘good’ if the estimated envelope did not deviate more than (approximately) ± 5 cm/s from the visually detectable envelope on at least 90 % of the recording duration. An envelope was categorized as ‘acceptable’ if the deviation was below ± 10 cm/s, and as ‘bad’ otherwise (see Fig. 10). This coarse (and subjective) evaluation metric allowed us to assess the performance of the envelope tracing algorithm on the full duration of 4 h and 40 min of TCD recordings.

### C. Algorithm Evaluation

To assess the performance of the proposed algorithm, we performed a quantitative evaluation, where we compared manually with algorithmically traced spectrogram envelopes. In addition to the comparison with manual tracings, we compared our algorithm to the MTCM algorithm [32], [33]. The key idea of the MTCM is to estimate the maximal frequency ($\mathrel {\widehat {=}}$ velocity) by comparing the spectral power in each frequency bin, starting from the high frequency end (noise floor $N_{0}$), to an empirically determined threshold $T_{{\mathrm {MTCM}}}$ that is held constant on a whole spectrogram segment and that depends on the SNR of the spectrogram. For the MTCM, we manually adapted this threshold to obtain a maximal blood flow velocity waveform whose visually-assessed quality was as high as possible. Due to the human-in-the-loop, this version of the MTCM, which we denote by MTCM++, is superior to the classical MTCM.

#### 1) Quantitative Evaluation Metrics

We define the error signal

\begin{equation*} e[n] = v_{{\mathrm {algo}}}[n] - v_{{\mathrm {man}}}[n],\tag{3}\end{equation*}

where $v_{{\mathrm {algo}}}$ is the estimated maximal flow velocity waveform, $v_{{\mathrm {man}}}$ the manually traced maximal flow velocity, $n = 1, \ldots, N$ the sample index, and $N$ corresponds to the total number of samples. The bias is defined as

\begin{equation*} m_{e} = \frac {1}{N} \sum _{n=1}^{N} e[n]\tag{4}\end{equation*}

and the variance of the error as

\begin{equation*} \sigma _{e}^{2} = \frac {1}{N} \sum _{n=1}^{N} \left ({e[n] - m_{e} }\right)^{2}.\tag{5}\end{equation*}

In addition to the absolute error, we computed the relative error that we defined as

\begin{equation*} r_{e} = \frac {\sum _{n=1}^{N} e[n]}{\sum _{n=1}^{N} v_{{\mathrm {man}}}[n]}.\tag{6}\end{equation*}

Since the peak-systolic velocity is an important quantity, e.g., used in the computation of the pulsatility and resistivity index and for the detection of vasospasms, we furthermore evaluated the absolute and relative differences in peak-systolic velocities between the manual tracing and the algorithmically obtained peak-systolic velocities. We define the peak difference as

\begin{equation*} \Delta ^{{\mathrm {peak}}}[k] = v_{{\mathrm {algo}}}^{{\mathrm {peak}}}[k] - v_{{\mathrm {man}}}^{{\mathrm {peak}}}[k],\tag{7}\end{equation*}

where $v^{{\mathrm {peak}}}[k]$ is the peak-systolic velocity of the $k$-th heart beat. Thereby, we can define the following error metrics

\begin{equation*} m_{\Delta ^{{\mathrm {peak}}}} = \frac {1}{\# {\mathrm {beats}}}\sum _{k=1}^{\#{\mathrm {beats}}} \Delta ^{{\mathrm {peak}}}[k].\tag{8}\end{equation*}

and

\begin{equation*} \sigma _{\Delta ^{{\mathrm {peak}}}}^{2} = \frac {1}{\# {\mathrm {beats}}}\sum _{k=1}^{\#{\mathrm {beats}}} (\Delta ^{{\mathrm {peak}}}[k] - m_{\Delta ^{{\mathrm {peak}}}})^{2}.\tag{9}\end{equation*}

and

\begin{equation*} r_{\Delta ^{{\mathrm {peak}}}} = \frac {\sum _{k=1}^{\# {\mathrm {beats}}} \Delta ^{{\mathrm {peak}}}[k]}{\sum _{k=1}^{\# {\mathrm {beats}}} v_{{\mathrm {man}}}^{{\mathrm {peak}}}[k]}.\tag{10}\end{equation*}

Note that for being able to compare estimates of positive flow velocities, as they occur in the MCA, with negative flow velocities, as they occur in the ICA, we flipped the sign of the negative flow velocities.

## IV. Results

In the following, we report the performance of our algorithm in terms of the quantitative and qualitative performance metrics outlined above, and also provide estimates of its runtime.

### A. Quantitative Evaluation Results

In each of the 32 recordings (16 recordings from healthy patients and 16 from neurocritical care patients), we manually traced a spectrogram segment containing 30 heart beats and computed the error metrics for our algorithm and the MTCM++ algorithm. As the fixed parameter MTCM failed on a large number of recordings (see Table 3), we did not perform a quantitative evaluation for this algorithm. When the fixed parameter MTCM fails on a recording, usually the returned maximal flow velocity is either almost constant and at the maximally measurable flow velocity, or if the algorithm is not sensitive enough, around zero. Table 1 and 2 summarize the computed performance metrics for the healthy subject population and for the neurocritical care patients, respectively. The average over the 32 recordings had a bias of $m_{e} = -0.7$ cm/s and a standard deviation of the error of $\sigma _{e} = 3.2$ cm/s. The deviation in peak systolic velocities was $m_{\Delta ^{{\mathrm {peak}}}} = -2.8$ cm/s.

**TABLE 1:** Quantitative Evaluation of Our Algorithm and the MTCM++ vs Manual Tracing on 16 Recordings From Healthy Subjects

Algorithm	$m_{e}$ [cm/s]	$\sigma _{e}$ [cm/s]	$r_{e}$ [%]	$m_{\Delta ^{{\mathrm {peak}}}}$ [cm/s]	$\sigma _{\Delta ^{{\mathrm {peak}}}}$ [cm/s]	$r_{\Delta ^{{\mathrm {peak}}}}$ [%]
Our	−1.1	2.9	−2.3	−3	2.9	−3.7
MTCM++	−1.7	4.3	−3.5	−4.2	5.3	−5.2

**TABLE 2:** Quantitative Evaluation of Our Algorithm and the MTCM++ vs Manual Tracing on 16 Recordings From Neurocritical Care Patients

Algorithm	$m_{e}$ [cm/s]	$\sigma _{e}$ [cm/s]	$r_{e}$ [%]	$m_{\Delta ^{{\mathrm {peak}}}}$ [cm/s]	$\sigma _{\Delta ^{{\mathrm {peak}}}}$ [cm/s]	$r_{\Delta ^{{\mathrm {peak}}}}$ [%]
Our	−0.3	3.4	−0.6	−2.5	3.5	−2.9
MTCM++	−3	4.7	−6.3	−4.6	4.3	−5.3

**TABLE 3:** Qualitative Evaluation of All 32 Recordings With Automatically Traced TCD Spectrograms

Algorithm	# good	# acceptable	# bad	# recordings
Our	29	2	1	32
MTCM	12	11	9	32
MTCM++	27	4	1	32

Note that our algorithm is calibration-free and therefore no manual parameter tuning was required to obtain the results in tables 1 and 2. As discussed above, for the MTCM++ in contrast, we manually adjusted the preset threshold on the pixel intensity until (if possible) we obtained an envelope we deemed of a high quality.

A representative spectrogram segment is shown in Figure 9, where the manual trace is shown in blue and the envelope obtained by the proposed algorithm in red. In this segment the bias is $m_{e} = -0.75$ cm/s, the standard deviation of the error is $\sigma _{e} = 2.42$ cm/s, and the average difference of the systolic peak is $m_{\Delta ^{{\mathrm {peak}}}} = - 5.3$ cm/s. In this example, we can see that the algorithm (slightly) underestimates the peak systolic flow velocity compared to the manual trace.

Due to the clinical importance of the peak systolic velocity, we performed a more detailed analysis of the estimation accuracy of our algorithm. For this, we compared the peak systolic velocity estimates of our algorithm with the manual traces consisting of 32 segments, each containing 30 beats. Figures 7 and 8 show the Bland-Altman plots for healthy and neurocritical care patients, where each circle denotes a single peak systolic velocity estimate. Positive peak systolic velocities stem from MCA, whereas negative peak systolic velocity stem from ICA measurements. Overall, the bias is very low and only a few beats show a significant deviation from the manually traced peak systolic velocities.

### B. Qualitative Evaluation Results

We visually assessed all of the 32 recordings with a total duration of about 4h and 40 min and scored the quality of the maximal blood flow velocity estimate of our algorithm, the fixed parameter MTCM, and the MTCM++ algorithm.

Table 3 shows the results of this qualitative evaluation. An example of the visual assessment is shown in Figure 10 that contains traces obtained by our algorithm (red) and from the MTCM (green). In general, for low SNRs and SNRs that change during one recording, the performance of the MTCM algorithm is impaired (Fig. 10 top). Note that with the MTCM version with a fixed parameter set, it is hardly possible to obtain a good tracing performance on all spectrograms. However, when manually adapting the parameters for each recording, we managed to obtain a good performance. Therefore, similarly to the proposed algorithm, the MTCM algorithm could be improved by a grid search around a suggested threshold with a subsequent selection of the trace with the highest signal quality.

### C. Run-Time

The current implementation of our algorithm uses five candidate grayscale thresholds for estimating the maximal flow velocity. It requires around 12 s to process a 1 minute long recording on a Macbook Pro with an iCore 7 processor, with all algorithmic steps implemented in MATLAB without particular attention paid to optimize the performance for real-time execution.

## V. Discussion

In this work, we have presented an envelope tracing algorithm for extracting maximal blood flow velocities from TCD spectrograms. The feedback loop between the spectrogram tracing and the signal quality assessment leads to an accurate estimation of the maximal flow velocity waveforms in different arteries, even in challenging scenarios with varying signal intensity and low SNR. Compared to physiology-agnostic edge detection or thresholding algorithms, the proposed approach uses physiological constraints to avoid spurious edges. The signal quality feedback loop provides the necessary adaptiveness required for the algorithm to detect maximal blood flow velocities in different vessels and helps with handling inter- and intra-subject variability, as shown for instance in Fig. 10. A further advantage of the current approach is that it returns a beat-by-beat signal-quality estimate alongside the detected maximal blood flow velocity waveform. Such a signal-quality metric is important if the flow velocity waveform or features thereof are to be passed to downstream processing or reasoning modules.

Our evaluation was aimed at cerebral vessels, primarily the MCA and ICA using one type of ultrasound device. In a future work, it would be interesting to evaluate the algorithm’s performance on other arteries, such as the umbilical artery as well as on different ultrasound machines.

From our anecdotal clinical experience, the peak systolic velocity seems to be underestimated in various commercial ultrasound systems as well as by algorithms from the academic literature, such as the MTCM. We assume that the main reason for the underestimation of peak systolic velocities is due to low-pass filtering employed for enhancing robustness of the envelope detection algorithms. Our algorithm has a systematic (but comparatively low) negative bias as well, as shown in Figures 7 and 8. This is mainly due to the low-pass filtering (median filtering of the spectrogram and low-pass filtering of the obtained envelope). Additionally, the signal quality algorithm, being partly based on template matching with an averaged beat, penalizes excessively variable waveforms and therefore might further increase the bias to overly smooth curves and thus rounded-off systolic peaks. Therefore, it would be worthwhile exploring additional features to be incorporated into the signal quality assessment. For example, a measure for the consistency and magnitude of the peak systolic velocity could further reduce the bias in the estimated peak systolic velocities.

The current implementation of our algorithm is tailored to offline processing. However, by working on a window-by-window basis and due to the processing time being much faster than the duration of the recording, the algorithm can be adapted for near real-time execution. A further speed-up can be achieved by using the segmentation threshold learned on past waveform segments and adapt the threshold only if the signal quality falls below some preset value.

**FIGURE 11.**
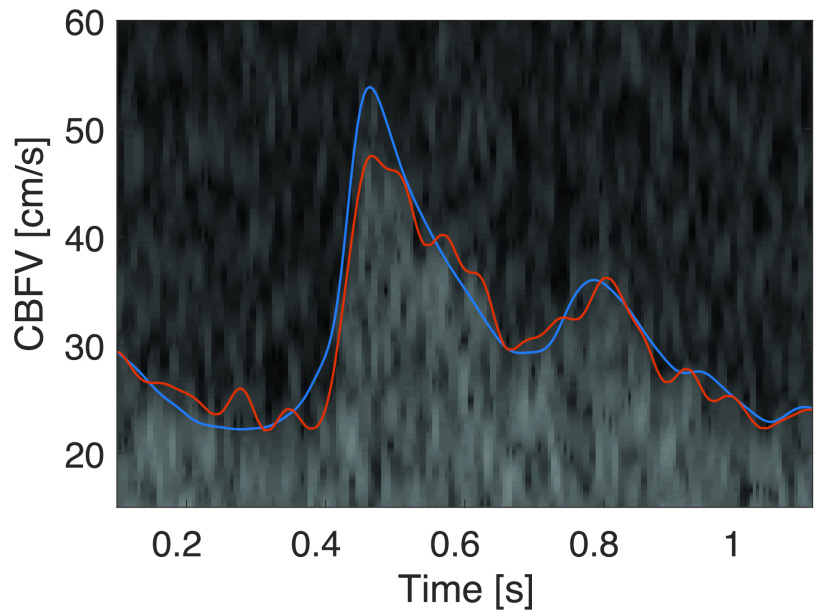
This figure shows the difficulty of finding a maximal blood flow velocity in low SNR spectrograms. Here, we can also appreciate the mismatch between the manual (blue) and algorithmic (red) tracing. Note that between 0.6s and 0.7s the manual tracing is arguably worse than the algorithmic trace.

The presented algorithm is able to recover the maximal flow velocity quite accurately when compared to manual tracings. It is important to note though that it is not always easy to visually detect the maximal flow velocity and in particular the peak-systolic velocity in a TCD spectrogram (see Fig. 11). Significant human inter-rater variability is often present [34], and spectral broadening as well as changes in the insonation angle complicate the determination of the maximal flow velocity. In addition, for guaranteeing a reliable human gold-standard for the quantitative evaluation, we only used segments in which it was possible to visually discern the envelope quite clearly. This excludes very low SNR segments, which are of little clinical value anyways. The subjectivity in the assessment is a limitation both for our visual assessment and for the quantitative evaluation of the retrieved spectrogram envelope compared to the manual trace. This being said, the residual errors of our algorithm seem of little clinical significance.

A comparison of the proposed approach to other algorithms in the literature is challenging. Algorithms such as [22] employ a shape-based approach using Gamma functions that are fit to umbilical Doppler echos. The output of the algorithm is an “average” cardiac cycle from which Doppler indices can be computed. Our goal was to obtain a sample-by-sample envelope for different vessels and therefore a comparison with [22] was not practical. Similarly, in [21] a supervised approach is presented that requires a subset of the data to be manually-traced from which a generic envelope template is learned. Since our goal was to have a fully unsupervised algorithm, a quantitative comparison was not deemed relevant. Nonetheless, the Kalman filter based envelope tracing from [21] encodes a similar idea to our approach for avoiding spurious edges, namely that the envelope trace is not allowed to change too much from one sample to the next. We finally opted for a comparison with the MTCM, since it is a simple and known method that is waveform-agnostic and fully unsupervised.

A recent and interesting approach to flow velocity estimation worth mentioning is [35]. There, the Doppler spectrum is assumed to be generated by a discrete number of cylindrical sample volumes with a specific flow profile (e.g., laminar). These assumptions yield a mathematical spectrogram model from which the maximal flow velocity can be obtained directly.

Irrespective of the employed algorithm, the difficulty in finding the maximal flow velocity in Doppler spectrograms has implications on derived indices such as the pulsatility index and other derived quantities such as ICP [8], [9], [11]. This uncertainty in the blood flow velocity waveform and the associated need for visual review and manual annotation of the waveforms to flag regions of sufficiently high signal quality is one of a series of challenges that need to be overcome in the translational effort to bring non-invasive ICP monitoring to the bedside. One way to tackle this challenge is to equip the flow velocity waveform with a signal quality index, such that in the downstream computation one can exclude low-quality segments.

Another way to increase clinical acceptance of TCD could be provided by new approaches that improve usability and signal quality, e.g., through electronic beamforming [36] or through robotically-driven TCD probes that automatically move the probe to find a positioning, where the received echo has a high signal intensity [37]. Integrating a signal quality assessment algorithm, such as the one proposed in this work, could potentially help to find a good orientation of the ultrasound probe.

## VI. Conclusion

In this work, we have proposed an algorithm for automatically tracing the maximal blood flow velocity in transcranial Doppler spectrograms. In addition to the maximal flow velocity, the algorithm provides an assessment of the signal quality on a beat-by-beat basis. By making both the code and the data publicly available we hope that the present work will be a useful resource in future experimental applications of TCD ultrasonography.

## Supplementary Material

10.21227/44mg-2965 TRANSCRANIAL DOPPLER ULTRASOUND DATABASE (PHILIPS CX50 DEVICE) Transcranial Doppler (TCD) echo data was recorded from healthy adults and neurocritical care adult patients. The insonated cerebral vessels were the middle cerebral artery (MCA) and the internal carotid artery (ICA). The ultrasound system used in this study was the Philips CX50. https://ieee-dataport.org/open-access/transcranial-doppler-ultrasound-database-philips-cx50-device
